# Changes of Sensory Quality, Flavor-Related Metabolites and Gene Expression in Peach Fruit Treated by Controlled Atmosphere (CA) under Cold Storage

**DOI:** 10.3390/ijms23137141

**Published:** 2022-06-27

**Authors:** Hongru Liu, Hui He, Chenxia Liu, Chunfang Wang, Yongjin Qiao, Bo Zhang

**Affiliations:** 1 Crop Breeding & Cultivation Research Institute, Shanghai Academy of Agricultural Sciences, Shanghai 201403, China; hear2008dream@163.com (H.L.); hh18536060094@163.com (H.H.); LW5715lcx@126.com (C.L.); fhwcf@126.com (C.W.); 2 Laboratory of Fruit Quality Biology Zhejiang Provincial Key/Laboratory of Horticultural Plant Integrative Biology, Zhejiang University, Hangzhou 310058, China

**Keywords:** peach, controlled atmosphere, chilling injury, aroma volatiles, sensory quality, gene expression

## Abstract

Controlled atmosphere (CA) has been used to alleviate chilling injury (CI) of horticultural crops caused by cold storage. However, the effects of CA treatment on peach fruit sensory quality and flavor-related chemicals suffering from CI remain largely unknown. Here, we stored peach fruit under CA with 5% O_2_ and 10% CO_2_ at 0 °C up to 28 d followed by a subsequent 3 d shelf-life at 20 °C (28S3). CA significantly reduced flesh browning and improved sensory quality at 28S3. Though total volatiles declined during extended cold storage, CA accumulated higher content of volatile esters and lactones than control at 28S3. A total of 14 volatiles were positively correlated with consumer acceptability, mainly including three C6 compounds, three esters and four lactones derived from the fatty acid lipoxygenase (LOX) pathway. Correspondingly, the expression levels of genes including *PpLOX1*, hyperoxide lyase *PpHPL1* and alcohol acyltransferase *PpAAT1* were positively correlated with the change of esters and lactones. CA elevated the sucrose content and the degree of fatty acids unsaturation under cold storage, which gave us clues to clarify the mechanism of resistance to cold stress. The results suggested that CA treatment improved sensory quality by alleviating CI of peach fruits under cold storage.

## 1. Introduction

Peach (*Prunus persica* L. batch) is a world-wide economic fruit with important commercial value [1]. However, its climacteric character causes it to perish and deteriorate during postharvest storage at room temperature. Cold storage can effectively inhibit physiological metabolism, which widely occurs during postharvest storage and cold-chain transportation of horticultural crops. However, peach fruit is sensitive to cold storage, especially at the mid-temperature (2.2–7.6 °C) [2,3]. Serious of symptoms have occurred when peach suffer from chilling injury (CI), including flesh browning, defect of ethylene synthesis, and loss of fruit scent and flavor [2,4,5]. Notably, CI symptoms mainly manifest and develop seriously during shelf-life after cold storage, though the physiological disorder is caused by cold storage [3,6,7]. Consumer dissatisfaction on postharvest fruit quality in last decades is mainly caused by the loss of fruit aroma and flavor after cold storage [8,9,10,11,12]. Thus, the discovery of efficient postharvest technologies that can furthest extend storage life and maintain fruit quality is an urgent work.

Many attempts to alleviate the development of CI after cold storage have been reported in peach fruit, such as low temperature conditioning [13], near-freezing temperature storage [14], 1-methylcyclopropene [15,16,17], nitric oxide [18,19], methyl jasmonate [20,21,22]. Controlled atmosphere (CA) was performed by mediating the atmosphere pressure of the storage microenvironment, mainly including low O_2_ and high CO_2_ pressure [23,24,25]. Different pressures of O_2_ and CO_2_ could induce different physiological metabolism such as anaerobic respiration [26], off-flavor products [27], superficial scald incidence [28], fruit softening [9]. The usual gas pressure was 3–5% CO_2_ and 1–2% O_2_ in traditional CA treatments [24,29]. Meanwhile, recent studies have shown that higher CO_2_ pressure could alleviate CI symptoms more efficiently than traditional treatments. For instance, a combination of 10% CO_2_ + 10% O_2_ could reduce CI in nectarines under 4 weeks of cold storage [23]. CA treatments have been developed to prolong storage life of many horticultural crops, including modified atmosphere with different air permeability package, dynamic controlled atmosphere storage, initial low oxygen stress atmosphere, and ultralow oxygen treatments [17,24,26,30]. The suitable atmosphere proportions and application modes are different for different horticultural crops and the different varieties [24,26]. For example, the ultralow oxygen condition of 0.8–1.2% O_2_ and 2–3% CO_2_ were mainly used to preserve ‘Gala’ apples not suitable for other apple varieties [17]. In summary, CA treatments have been widely used in the preservation of apples [24], litchi [28], broccoli [31], green asparagus [30], peaches and nectarine [5,15]. CA treatment can effectively delay senescence, alleviate chilling injury, improve flavor quality of horticultural crops through enhancing the antioxidant capacity, improve sucrose content, influence respiratory pathway, and so on [5,24,29,31,32,33,34]. For instance, core browning of ‘Yali’ pear was alleviated by reducing PPO activity, phenolic content and genes expression of *PbPAL1*, *PbPAL2* and *PbPPO1* under modified atmosphere packaging (MAP) with 10-µm-thick low-density polyethylene [33]. However, the effect of CA treatment on the change of peach fruit volatiles and the relationship to sensory quality after cold storage continue to be gaps in our knowledge.

Fruit flavor is influenced by hundreds of volatiles, soluble solids and some other unknown metabolites [35,36,37]. The mixtures of volatiles and non-volatile metabolites present a challenge when it comes to evaluating the key contributors of fruit flavor quality. Peach fruit contained more than 100 volatiles, including C6 aldehydes and alcohols, esters, lactones, terpenoids and so on, mainly derived from fatty acids, amino acids and carbohydrate [38,39,40]. Generally, C6 compounds contribute green sensory notes and esters, while lactones combined with some terpenoids provide fruity and floral notes of ripe peaches [6,40]. For instance, esters, γ-decalactone, and β-ionone were presumed as key contributors to peach fruity aroma among the Four National Traditional Famous Peaches of China [41]. Loss of fruity flavor after postharvest cold storage was mainly due to impairment of the key aroma volatile synthesis [6,7]. Peach fruit accumulated significant low content of hexyl acetate, (*E*)-2-hexenyl acetate, (*Z*)-3-hexenyl acetate and lactones suffering from CI and (*Z*)-3-hexenyl acetate showed a positive relationship with consumer liking [3,6,10,42]. Though amounts of volatile compounds have been identified in peach fruit, the interaction roles between volatiles and other metabolites in contributing to fruit flavor remain unclear. Soluble sugars in peach fruit are mainly composed of sucrose, glucose, fructose and sorbitol. Generally, sucrose approximately occupied 40–85% of total soluble sugars, while glucose and fructose only accounted for 10–25% together [43]. As peach fruit ripened after postharvest, sucrose declined slightly while the content of glucose and fructose tended to increase during cold storage and subsequent shelf-life. Sucrose was beneficial for enhancing fruit tolerance to cold stress [44]. Organic acids of ripe peach fruit are mainly composed of malic acid, citric acid and quinic acid, with malic acid being the dominant proportion [43]. Meanwhile, the organic acids are respiratory substrates, showing a declining trend during postharvest cold storage [43,45]. Thus, the determination of the chemical components of peach fruit is important but not enough for comprehensively evaluating fruit flavor quality. Chemical determination, sensory analysis, and molecular analysis need to work together to clarify the key contributors to fruit sensory quality formation, such as chemical metabolites, genes, proteins and so on.

As mentioned above, the aroma volatiles derived from the fatty acids oxidation pathway are important contributors to fruit flavor quality, which are significantly reduced by CI after postharvest cold storage [3,6]. Thus, the genes involved in the metabolism of the LOX pathway with precursors of linoleic and linolenic need to be clarified. Briefly, LOX and HPL firstly transform linoleic and linolenic acids to hexanal and hexenal many times over. Then the aldehydes can be converted to the corresponding alcohols by ADH. The final esters are catalyzed by AAT with the linkage of an acyl moiety and an alcohol acceptor [46]. In addition, the lactone could also be synthesized by enzyme AAT among peach varieties [47,48]. In summary, esters and lactones derived from the fatty acid LOX pathway are positively correlated with the expression of the specific gene family members of *PpLOX1*, *PpLOX3, PpHPL1* and *PpAAT1* during fruit ripening and shelf-life after cold storage [6,46]. Meanwhile, the expression levels of the specific genes of the LOX pathway were generally inhibited in CI peach fruit after postharvest cold storage [3,6]. Thus, the key genes expression level could significantly influence the content of the aroma volatiles and the fruit flavor quality.

In summary, CA treatment could effectively alleviate CI caused by postharvest cold storage in many horticultural crops, though the suitable proportions of gas varied differently [24,29]. However, comprehensive analysis of the volatile compounds and non-volatile metabolites that contribute to peach fruit flavor quality is still necessary. In the present study, a suitable CA treatment for peach fruit was applied to explore effects on alleviating CI and maintaining fruit flavor quality. In addition, the key aroma volatiles and metabolites contributing to flavor quality were screened out and correlated with consumer sensory perception in peach fruit after postharvest cold storage. The specific genes exhibited similar increasing patterns for accumulating the aroma volatiles derived from the LOX pathway under CA treatment during shelf-life after postharvest cold storage.

## 2. Results

### 2.1. Influence of CA Treatment on Firmness, Ethylene Emission, Total VOC Production and Flesh Browning of Peach Fruit after Cold Storage

**Figure 1:**
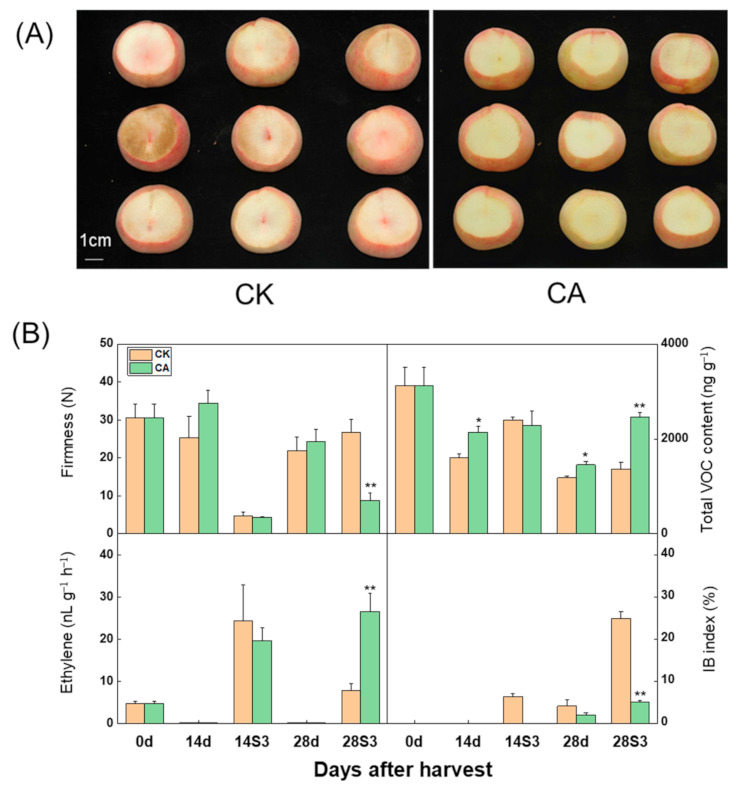
Physiological changes of peach fruit under CA treatment during the cold storage and shelf-life. (**A**) Photos of peach fruit under CA treatment after 28 d cold storage with 3 shelf-days at 20 °C; (**B**) Changes of the firmness, ethylene emission rate, total volatile organic compounds (VOC) and IB index of peach fruit under CA treatment. Data are expressed as the means ± standard error of three biological replicates, the * indicated the statistical significance of the two samples with the unpaired Student’s *t*-test (* p < 0.05, ** *p <* 0.01. 28S3CK: the first number represent days after postharvest storage, S, means shelf-life, 3 means the days during shelf-life.

The combination of 5% O_2_ + 10% CO_2_ was used to analyze effects of CA treatment on peach fruit quality under cold storage plus subsequent shelf-life. The picture shows that severe flesh internal browning occurred in control during three shelf-days at 20 °C after 28 d cold storage (28S3), while CA treated fruits maintained fresh flesh well (Figure 1A). The IB index was used to represent the degree of flesh browning, which was one typical CI symptom in peach fruits [2]. The IB index increased from lower than 10% to higher than 25% as storage was prolonged from 14 d to 28 d plus three shelf-days (Figure 1B). CA treatment kept the IB index lower than 10% even at 28S3 (Figure 1B). Normal ripening after cold storage with ethylene release peak and firmness softening was important for peach fruit quality [6]. Fruits soften normally with firmness decreasing lower than 10 N and releasing high ethylene peak at 14S3 with little CI index (Figure 1B). Meanwhile, the control fruits that suffered from severe CI had their softening capacity retarded and maintained their firmness at 30 N and had a small detection of ethylene emission at 28S3 (Figure 1B). On the other hand, normal ripening was observed under CA treatment at 28S3, with firmness decreased to 7 N and a significantly higher ethylene released peak than control (Figure 1B). The aroma volatile was one of the main contributors to fruit flavor quality and was easily influenced by CI [4]. CA treatment significantly delayed the decline of the peach fruit total VOC, especially at 28S3 with nearly 50% increase compared with control (Figure 1B, Table S1). In summary, the specific CA treatment efficiently alleviated the development of peach fruit CI after cold storage and maintained fruit quality as normal ripening during shelf-life.

### 2.2. Sensory Quality Evaluation of Peach Fruit during Shelf-Life after Cold Storage

**Figure 2:**
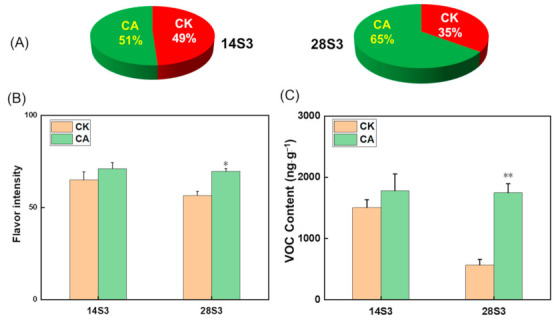
Sensory evaluation and VOC content of peach fruit during three shelf-days after cold storage. (**A**) Aroma preference of the fruit; (**B**) Flavor intensity of the fruit; (**C**) Total content of the key volatile compounds. The sensory evaluation of peach fruit was performed by 30 panelists. The key volatile compounds were listed in Table S1 with bold highlighted. Data are expressed as the means ± standard error of three biological replicates, the * indicated the statistical significance of the two samples with the unpaired Student’s *t*-test (* *p <* 0.05, ** *p <* 0.01).

To evaluate the effect of CA treatment on sensory quality, sensory analysis was performed by a sensory panel. Panelists showed no obvious preference between control and CA treated fruits at 14S3 with 51% versus 49%, whereas they showed more preference for CA treatment fruits compared to control at 28S3 with 65% versus 35% (Figure 2A). Correspondingly, the flavor-liking intensity showed no significant difference at 14S3, while a significant higher score for CA treatment than control as storage was prolonged to 28S3 (Figure 2B). Peach fruit contained more than 100 volatile compounds, and 21 kinds of them positively contributed to scent with odor activity values higher than 1 [38,40]. The content of the volatile, which were presumed as beneficial contributors to peach flavor quality [38,40], dramatically declined about 70% at 28S3 in control, while CA treatment maintained the specific VOC content steadily (Figure 2C, Table S1).

Three main organic acids and soluble sugars were detected under cold storage plus shelf-life (Table 1). Sucrose occupied nearly 80% of total soluble sugars in peach fruit and decreased from 151.1 mg × g^−1^ to 104.80 mg × g^−1^ when storage was prolonged to 28S3 in control (Table 1). CA treatment significantly delayed sucrose content decline, with only a 10% reduction compared with 0 d at 28S3 (Table 1). Inversely, the content of glucose and fructose increased to 2.3-fold and 2.8-fold, respectively, compared with 0 d in control, while CA treatment accumulated only 30% of control at 28S3 (Table 1). Thus, CA treatment accumulated a higher content of total soluble sugars at 14S3 compared with control, while it significantly inhibited accumulation more than control at 28S3 (Table 1). Malic acid occupied nearly 66% of the total acids in peach fruit, and all three acids exhibited a decreasing trend during storage. Although CA treatment showed no significant influence on the accumulation of organic acids, the total content declined more slowly than control during cold storage and shelf-life (Table 1). In summary, CA treatment efficiently maintained the sensory quality of peach fruit and delayed the decline of VOC content and organic acids. Meanwhile, CA treatment elevated sucrose content and inhibited the increase of glucose and fructose compared with control during cold storage and shelf-life, especially at the later stage of storage.

### 2.3. Production of Peach Fruit VOC under CA Treatment during Shelf-Life after Cold Storage

**Figure 3:**
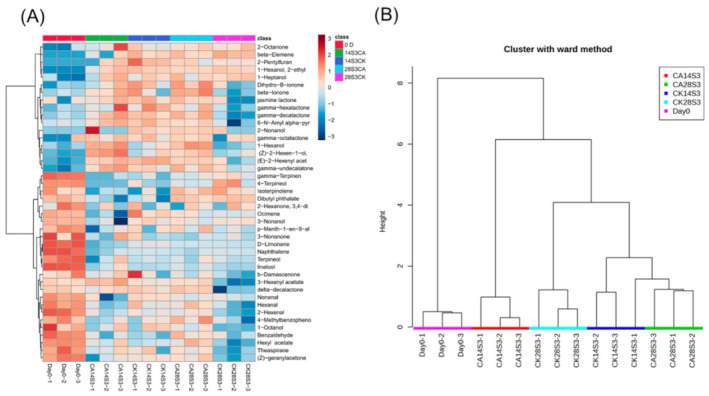
Cluster analysis of peach fruit volatiles during shelf-days after cold storage. (**A**) Heatmap of the peach fruit volatiles; (**B**) Cluster analysis of the peach fruit volatiles. CA28S3-1: the second number represents days after postharvest storage, S, means shelf-life, 3 means the days during shelf-life, the last number means the replicates.

To measure effects of CA treatment on the production of VOC, cluster analysis with Ward’s method was performed during cold storage plus shelf-life (Figure 3 and Supplementary Figure S1). The heatmap of the total VOC showed the Day 0 sample clustered independently with rich amounts of terpenoids and aldehydes, which decreased dramatically with storage duration (Figure 3A). Both CA and control fruits produced little esters and lactones under cold storage, which were positively correlated with fruit ripening (Figure S1A). Generally, forty-two kinds of volatile compounds were detected among samples (Table S1), and only six compounds showed no significant difference among the samples, calculated with One-way ANOVA (Figure S1B). CI fruit of CK28S3 lost most of the volatile compounds compared with CA treatment, including C6 aldehydes, alcohols, aliphatic esters, lactones and C_13_ norisoprenoid volatiles (Figure 3A). Corresponding to the difference in volatiles, the fruit samples exhibited similar cluster patterns (Figure 3B). The Day 0 sample was separated alone from the others, and CA28S3 clustered together with CK14S3 at the other side. Meanwhile, the CI fruit of CK28S3 was independently separated from the CK14S3 cluster, located between CA14S3 and the CK14S3 cluster (Figure 3B). In summary, CA treatment exhibited an obvious influence on the production of volatiles during cold storage and subsequent shelf-life. Heatmap cluster analysis separated specific CI fruit of CK28S3 from the healthy fruit well.

### 2.4. Clarifying Peach Fruit VOC Roles in Influencing Sensory Quality

**Figure 4:**
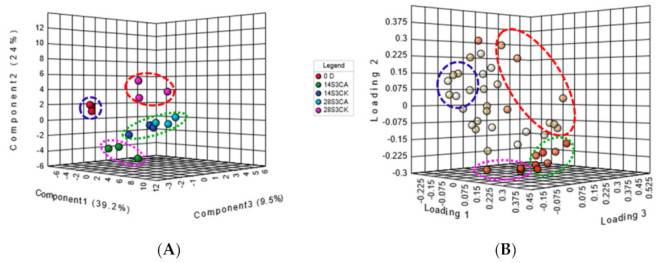
PLS-DA analysis of peach fruit VOC under CA treatment during shelf-life. (**A**) PLS-DA score plot of peach fruit VOC, the left one is the subfigure A; (**B**) Loading plot of peach fruit VOC with PLS-DA model, the right one is the subfigure B. The volatile compounds circled in different color circles were listed in Table S1.

CA treatment influenced both the consumer preference and volatiles content of peach fruit, while the interactional roles between them was not clear. Therefore, a partial least squares-discriminant analysis (PLS-DA) model was used to study the differences of the samples and clarify the characterized variables (Figure 4). PLS-DA analysis showed that the fruit samples were divided into four groups (Figure 4A). 14S3CK and 28S3CA clustered together and separated from 14S3CA, 0 D and 28S3CK samples. The total score of PC1, PC2 and PC3 reached 72%, which could explain the differences of the samples well (Figure 4A). The clustered pattern of the fruit samples was consistent with the PCA analysis result (Figure S2A). Cluster of 28S3CA was located on quadrant of negative PC1, PC2 and positive of PC3, cluster 28S3CK was located on quadrant of negative of PC1 and positive of PC2 and PC3, cluster 14S3CA was located on quadrant of negative PC1, PC2 and PC3 and OD sample was located on quadrant of negative of PC1, PC3 and positive of PC2 (Figure 4A). To clarify variables further, the loading plot of the PLS-DA exhibited four distinct clusters consistent with the PLS-DA score plot among three loadings (Figure 4B). The green circle of the loading plot included esters, lactones, and partial C_13_ norisoprenoid volatiles, and three main lactones in the pink red circle characterized the sample of 14S3CA (Figure 4B, Table S1). Meanwhile, six VOCs including terpenoids were predicted as the contributed variables for 28S3CK in the red circle, and seven VOCs of terpenes in the blue circle were presumed as the important variables for 0 D sample (Figure 4B, Table S1). All the loading plot compounds were consistent with the bioplot of PCA (Figure S2B). Variable Importance for the Projection (VIP) score could be used to measure the interpret ability and influential intensity of the VOC concentration on differences of samples. A total of 23 kinds of VOCs were selected as crucial VOCs in distinguishing different sample clusters by VIP (VIP > 1) and coefficients (>25) score (Figure S3). In summary, both the PLS-DA and PCA analysis could help to distinguish the samples among four main clusters. The loading plot of the two models indicated to us the characterized VOCs that might influence peach fruit sensory quality during shelf-life after cold storage.

**Figure 5:**
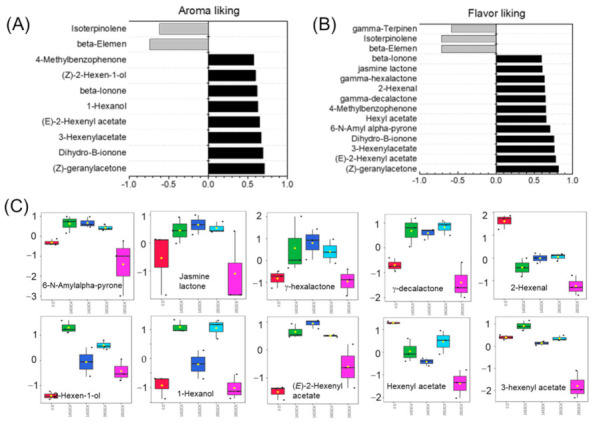
Identified peach fruit volatiles shown significant correlation with aroma- and flavor-liking during CA storage. (**A**) Volatiles correlated with aroma-liking; (**B**) Volatiles correlated with flavor-liking; (**C**) Content of the positively correlated volatiles.

To further ascertain the determined VOCs of sensory quality, the correlation relationship between the presumed VOCs and aroma- and flavor-liking were analyzed (Figure 5). A total of fourteen VOCs were positively correlated with aroma- and flavor-liking, and three terpenoids were negatively correlated with sensory quality (Figure 5A,B). Interestingly, ten of the positively related VOCs were all derived from fatty acid oxidation pathway, including C6 aldehydes, alcohols, three main esters and four lactones. The other four VOCs were three C_13_ norisoprenoids and one ketone (Figure 5A,B). Lactones and esters were accumulated higher in healthy fruit during shelf-life compared with CI fruit of 28S3CK (Figure 5C). CA treatment accumulated relative high content of two C6 alcohols during shelf-life (Figure 5C). Interestingly, only the C6 aldehyde of 2-Hexenal accumulated highest content at 0 D and might result in the lowest content of downstream products of 2-Hexen-1-ol and (*E*)-2-Hexenyl acetate (Figure 5C). In addition, three main C_13_ norisoprenoids and one ketone showed a high accumulation at 28S3CA, and the content were dramatically decreased when CI occurred (Figure S4A). Peach fruit had an unpleasant smell and a disgusting taste at 28S3CK with severe CI. Hence, the three terpenoids might be presumed to be the bad flavor contributors in peach fruit quality for their high accumulation at 28S3CK (Figure S4B).

### 2.5. Effect of CA Treatment on Content of Fatty Acids and Derived VOCs

**Figure 6:**
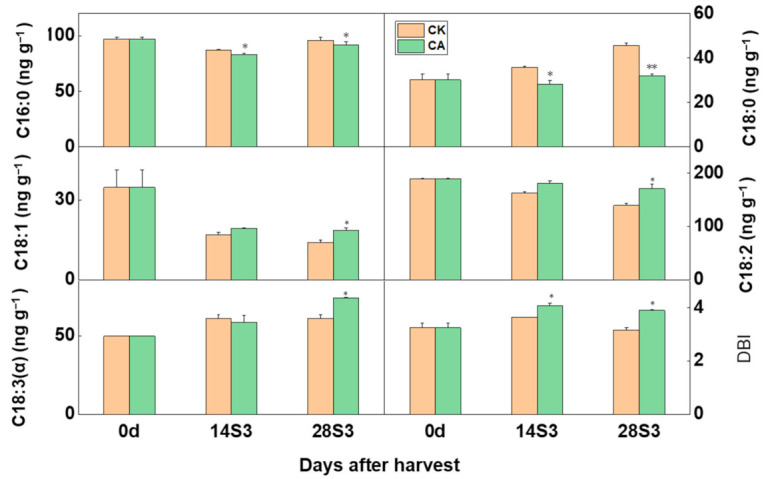
Determination of the fatty acid under CA treatment during the Shelf-life after cold storage. Data are expressed as the means ± standard error of three biological replicates and the * indicated the statistical significance of the two samples with the unpaired Student’s *t*-test (* *p* < 0.05, ** *p* < 0.01).

A total of five kinds of fatty acids were detected in peach fruit, including two saturated fatty acids of palmitic acid (C16:0) and stearic acid (C18:0) and three unsaturated fatty acids of oleic acid (C18:1), linoleic acid (C18:2), and linolenic acid (C18:3) (Figure 6). All fatty acids showed an increased trend during shelf-life after postharvest cold storage except for oleic acid (Figure 6). For peach fruit, linoleic acid was the most dominant fatty acid, occupying nearly 50% of the total fatty acids content (Figure 6). CA treatment significantly decreased content of saturated fatty acid and elevated unsaturated fatty acid accumulation during shelf-life after postharvest cold storage. Thus, the double bound index (DBI) of peach fruit under CA treatment was significantly higher than control during shelf-life after cold storage (Figure 6). Transformation between the saturation and unsaturation of fatty acids was controlled by PpFADs and *PpFAD1* showed a high expression level under CA treatment (Figure S5). In summary, CA treatment increased the content of unsaturated fatty acids and decreased the saturated fatty acids, resulting in the higher DBI than control.

**Figure 7:**
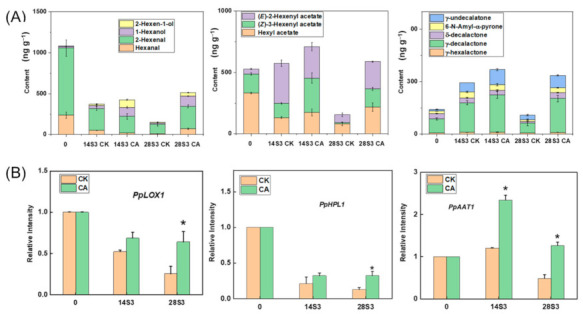
Change of the VOC and related genes expression derived from fatty acid LOX pathway during shelf-life. (**A**) Content of peach fruit VOC; (**B**) Relative intensity of gene expression. All data were from three biological replicates. Data are expressed as the means ± standard error of three biological replicates, and the * indicated the statistical significance of the two samples with the unpaired Student’s *t*-test (* *p <* 0.05).

The aroma volatiles derived from fatty acids were important contributors of peach fruit scent and flavor [40,46]. Fatty acids were firstly oxidized by PpLOX1, and then the hydroperoxide products were transformed into aldehydes by PpHPL1 [46]. The aliphatic esters in peach fruit could be synthesized by PpAAT1 with corresponding alcohols and acids [46]. Meanwhile, PpAAT1 also responded to the accumulation of lactones among peach varieties [47,48]. The content of aldehydes, esters and lactones decreased dramatically at 28S3CK due to CI (Figure 7A). For instance, total content of C6 compounds decreased from 1000 ng × g^−1^ to 100 ng g^−1^ at 28S3CK when compared with that at 0 d. Correspondingly, the esters content declined 80% compared with 0 d fruit in control. On the contrary, CA treatment accumulated nearly a 3-fold content of esters and a 2.2-fold content of lactones compared with control at 28S3 (Figure 7A). The accumulation of lactone was positively correlated with fruit ripening during shelf-life, and was dramatically inhibited by CI [6]. Content of lactone reached to 2.5-fold of 0 D at 14S3CA, in accord with the significantly higher expression of *PpAAT1* (Figure 7B). Meanwhile, CA treatment obviously induced expression of *PpLOX1*, *PpLOX3*, and *PpHPL1* from the fatty acid LOX pathway at 28S3 (Figure 7B and Supplementary Figure S5). In summary, CA treatment alleviated the severity of CI by increasing the DBI of the fatty acids. Further, the higher expression levels of key genes from the LOX pathway synthesized the higher content of esters and lactones than control under CA treatment during shelf-life after postharvest cold storage.

### 2.6. Analysis of Full-Data with K-Means and PCA Model during Shelf-Life after Cold Storage

**Figure 8:**
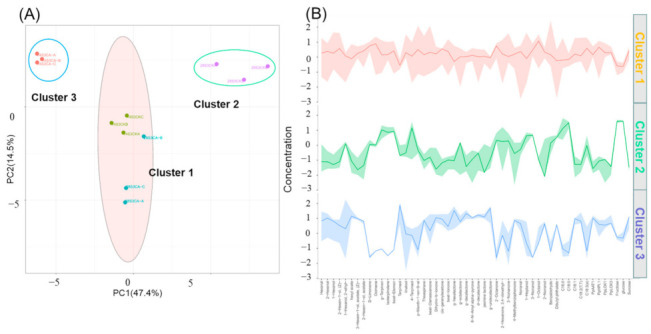
Cluster analysis of volatiles production, fatty acids, soluble sugars and genes expression during shelf-life after cold storage. (**A**) Cluster analysis of fruit samples; (**B**) Variation trend of characters involved in cluster. 28S3CK: the first number represents days after postharvest storage, S, means shelf-life, 3 means the days during shelf-life, the last letter means the biological replicates.

To clarify effects of CA treatment on peach flavor quality comprehensively, K-means and PCA model were used to perform cluster analysis with full-data. As shown in Figure 8, the fruit samples were distinctly divided into three clusters, and sample 14S3CK clustered together with 28S3CA, which indicated the high similarity of the two samples. Cluster 1 was located on the middle of PC1, while cluster 2 and cluster 3 were located on the opposite sides of PC1, respectively (Figure 8A). The total score of PC1 and PC2 explained about 62% of the total variables with characterization of volatiles, soluble sugars, fatty acids and genes expression during shelf-life after postharvest cold storage (Figure 8A). Correspondingly, the full characterized data showed three changeable trends, and cluster 3 of 14S3CA and cluster 2 of 28S3CK fluctuated visibly compared with cluster 1 (Figure 8B).

**Figure 9:**
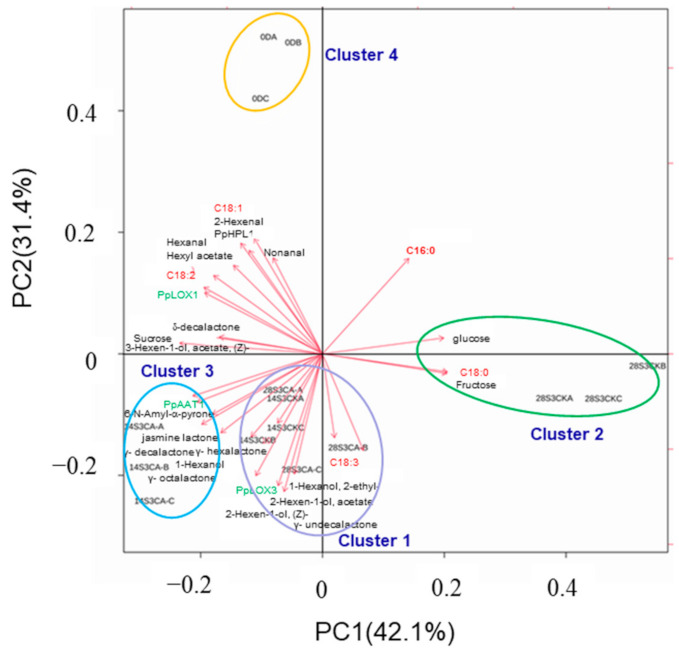
Biplot for the first two principal components of PCA for production of volatiles, fatty acids, soluble sugars and genes expression derived from the LOX pathway in peach fruit during shelf-life after cold storage. 28S3CK-A: the first number represents days after postharvest storage, S means shelf-life, 3 means the days during shelf-life, and the last letters means the replicate groups.

Further, PCA biplot analysis exhibited the contributed key factors under CA treatment clearly (Figure 9). PC1 and PC2 accounted for 73.5% of the total variability with characterization of volatiles, fatty acids, soluble sugars and genes expression (Figure 9). All samples were clustered into four groups, and samples of 14S3CA and 28S3CK were divided into cluster 3 and cluster 2, respectively, located on the opposite sides of PC1. Cluster 1 consisted of samples of 14S3CK and 28SS3CA, and mainly were located between cluster 2 and cluster 3 of PC1 (Figure 9). Notably, the CI fruit of Cluster 2 was located far away from the initial fruit of cluster 4, with little aroma-related volatiles characterized except for the glucose, fructose and C18:0 (Figure 9). On the other hand, normal ripening fruit of cluster 1 and cluster 3 was characterized by esters, lactones, fatty acids precursors of C18:3 and related genes of *PpLOX3* and *PpAAT1* from the LOX pathway (Figure 9). The initial fruit of cluster 4 was located on the upper left quadrant of the score plot alone, which was characterized by C6 aldehydes, partial esters and lactones, fatty acids of C18:2, C18:1 and genes of *PpLOX1*, *PpHPL1* from the LOX pathway (Figure 9). The cluster pattern of the full-data was consistent with Figure 8 and Figure 4. In summary, the k-means and PCA analysis exhibited key metabolites and genes influenced by CA treatment during shelf-life after postharvest storage.

## 3. Discussion

**Figure 10:**
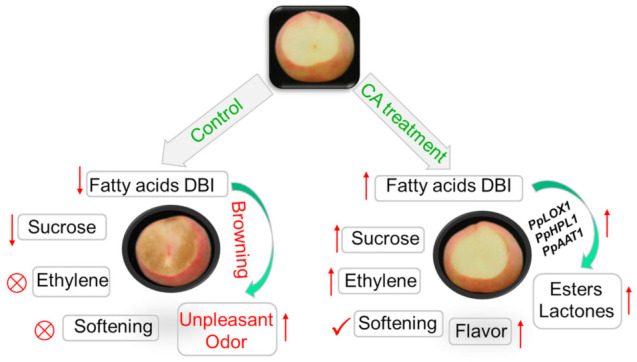
A proposed model of peach fruit biochemical and molecular change under cold storage plus subsequent shelf-life.

Peach fruit are easily perishable and deteriorate at room temperature, and cold storage is an effective way to prolong storage life [49]. However, the loss of fruit flavor quality after postharvest cold storage caused by CI has reduced consumer satisfaction for decades [10,50]. CA treatments have been widely used to extend storage life and alleviate CI of fruits [29]. However, the effects of CA treatment on the flavor quality of peach and the related key metabolites were not clear. In the present study, CA treatment could enhance peach fruit cold tolerance by elevating the DBI of fatty acids and sucrose content, and the effects of fatty acids and sucrose on cold resistance have been reported in many horticultural crops [51,52]. Thus, CA treatment maintains the normal ripening of peach fruit, with high ethylene release, softening capacity and high expression levels of related genes during shelf-life after postharvest cold storage (Figure 10). In addition, the high DBI of fatty acids supplied sufficient substrates for aroma volatile products derived from the fatty acid LOX pathway, which contributed importantly to fruit flavor quality and consumer acceptability (Figure 10).

Peach fruit endured cold temperature stress by mediating the series of physiological metabolism, such as soluble solids metabolism, conversion between saturated and unsaturated fatty acids, phytohormones signal transduction and the enhancing of antioxidant capacity [42,51,52]. In the present study, the specific CA treatment significantly alleviated CI of peach fruit at 28S3 with normal softening capacity, emission peak of ethylene, low IB index and high accumulation of VOC at 28S3 compared to control (Figure 1). Correspondingly, CA treatment fruit exhibited higher consumer satisfaction during shelf-life after cold storage (Figure 2). Fatty acids were important components of cell membrane and the high DBI of fatty acids improved the membrane mobility and enhanced resistance to chilling injury in peach fruits [22]. A certain level of endogenous ethylene or exogenous ethylene is necessary for nectarines normal ripening after cold storage [15,53].

Fruit flavor quality was a comprehensive character, including color, acids, sweetness, aroma, firmness and some other metabolites [8,35]. So far, more than 100 volatile compounds have been identified in peach fruit and most of them were easily damaged by cold storage [2,6,38]. Peach fruit flavor quality was generally related to the aroma volatiles, including C6 compounds, aliphatic esters, lactones and C_13_ norisoprenoids during fruit ripening and after postharvest cold storage [38,40]. CA treatment improved flavor quality of horticultural crops by elevating the content of aroma-related volatiles [5,23,26,29,32,50]. For instance, CA treatment accumulated higher levels of ethyl acetate with higher expression of *MdAAT1* induced by high ethylene in ‘Galaxy’ apples [17]. Peach fruit flavor quality was improved obviously under CA treatment; meanwhile, the total key volatile compounds were elevated higher than control at 28S3 (Figure 2C). The CI fruit of CK28S3 was separated distinctly from the healthy fruits including samples of CK14S3 and CA28S3 during shelf-life after cold storage (Figure 3). The cluster patterns among the fruit samples were consistent with the PLS-DA and PCA analysis results (Figure 4A and Figure S2). Generally, the characterized volatiles identified by the loading plot, VIP score, and coefficients index of PLS-DA mostly included C6 compounds, esters and lactones (Figure 4, Figures S2 and S3). Further, the linear regression between volatiles and sensory quality was calculated, and fourteen volatiles were characterized as the positive compounds, which mostly coincided with the previous characters (Figure 5). While flavor was perceived from the mixture of hundreds of metabolites, the clarification of the key contributors was important and challenging [35]. Many analysis attempts have been used to screen out the key characters from the large number of chemical compounds [8,11,36]. For instance, PCA model was used to evaluate effects of storage conditions on physiological and sensory quality of nectarine [49]. Recently, the gradient boosting machines and XGBoost were presumed as suitable models to predict sensory perceptions of fruit flavor among different varieties metabolites of tomato and blueberry [11,36]. The terpenoids of β-Elemene and isoterpinolene showed high correlation with CI fruit of 28S3CK, which indicated the off-flavor contributors to CI fruit (Figure 4, Figure S3 and Figure 5A).

The presumed aroma volatiles positively related to good flavor quality were mostly derived from the LOX pathway with substrates of linolenic and linoleic [6,46]. Unsaturated fatty acids were significantly induced by CA treatment at 28S3, while the saturated fatty acids increased in control (Figure 6). Thus, the high DBI of fatty acids increased the mobility of cell membrane, and reduced membrane damage when suffering from low temperature. C6 compounds showed a decreasing trend during shelf-life after cold storage, especially for CI fruits (Figure 7). Correspondingly, the expression levels of *PpLOX1*, *PpLOX3* and *PpHPL1* declined during shelf-life after storage (Figure 7 and Supplementary Figure S5). On the other hand, the content of esters and lactones increased during shelf-life after postharvest cold storage except for the CI fruit of 28S3CK, corresponding to the higher expression level of *PpAAT1* than control under CA treatment (Figure 7). The accumulation pattern indicated the ethylene was involved in the recovery of the fruity note aroma volatiles during shelf-life normal ripening after cold storage in peach. These results indicated that the accumulation of the aroma volatiles derived from the LOX pathway resulted from both the fatty acids substrates supply and the related key genes expression, including linoleic acid, linolenic acid, *PpFAD1*, *PpLOX1*, *PpLOX3*, *PpHPL1* and *PpAAT1.* Moreover, *PpAAT1* showed the high relationship to synthesis of esters and lactones, which was consistent with previous reports [6,47,48].

Soluble solids are not only a contributor of fruit flavor, but also an important indicator of CI in peach fruit [44]. A total of 23 metabolites were presumed as the important contributors to tomato consumer acceptability, and the content was usually decreased after cold storage [4,35,37]. Peach fruit showed a decreasing trend of sucrose with degradation and an increasing trend of glucose and fructose as postharvest ripening, especially for the CI fruit [45,49,51]. In the present study, CA treatment significantly delayed the degradation of sucrose and the increase of glucose and fructose when peach fruit were suffering from CI at 28S3 (Table 1). In addition, the content of organic acids was maintained steadily compared with control under CA treatment during shelf-life after postharvest cold storage (Table 1). Taken together, the soluble solids, aroma-related volatiles, fatty acids and related genes expression were characterized to distinguish the fruit samples, and the first two principles accounted for 73.5% of the distinguished results (Figure 9). Cluster 1 of 14S3CK and 28S3CA was located between cluster 3 of 14S3CA and cluster 2 of 28S3CK, characterized by the steady changeable trend of characteristics (Figure 8 and Figure 9). Thus, CA treatment could prolong peach fruit storage from 14 d to 28 d and maintain fruit quality well, which was consistent with previous reports in horticultural crops [26,29,54,55].

## 4. Materials and Methods

### 4.1. Plant Materials and Treatments

The peach (*Prunus persica* L. Batsch cv. Hujingmilu) is one typical white-fleshed melting peach, which tastes delicious and is planted widely in China. Peach fruit with uniform size was harvested at a commercial maturity from the standard orchard in the Nanhui district, Shanghai City, China. The fruits were immediately transported to the laboratory at a low temperature. Uniform fruits free of visual pathogen, pests and mechanical injury were carefully selected and divided into three groups randomly. About 20 fruits were sealed in one 50 L container with two pipelines mediating the gas proportion by O_2_, CO_2_ and N_2_, and were kept at 85–90% relative humidity at 0 °C. The CA treatment contained a 5% O_2_, 10% CO_2_ and 85% N_2_, while the control fruit was piped with dry air. These peach fruits were sampled at 14 d, 28 d plus 3 shelf-days at room temperature, respectively. Three fruits of each biological replicate were sampled with three biological replicates at each sampling point. The physiological data of firmness, ethylene emission rate and flesh internal browning were determined, and slices of flesh were frozen in liquid nitrogen and kept in -80 °C for further biochemical and molecular analysis.

### 4.2. Analysis of the Firmness, Ethylene, and Flesh Browning

The firmness of fruit was determined according to our previous report [46]. A texture analyzer (TA-XT2i Plus, Stable Micro system) equipped with a 7.9-mm diameter head was used to analysis fruit firmness. Two opposite sides of the fruit at the equator were used for firmness analysis with 1 mm × s^−1^ penetration rate, and the final penetration depth was 10 mm.

The measurement of fruit ethylene emission rate was performed as previously reported [46]. Briefly, an air-tight container (1.8 L) composed of polypropylene was used to seal two fruits of each biological replicate for 1 h at 0 °C or 20 °C (shelf-life). After sealing, 1 mL headspace gas was sampled to measure the ethylene production with a gas chromatography (Agilent Technologies 6890 N GC System, city, CA, USA), equipped with flame ionization detector (FID) at 230 °C [46]. The ethylene was separated by the column of GDX-502 (2 m × 3 mm i.d.), packed with a polymer of styrene and divinylbenzene. The carrier gas was nitrogen with a flow rate of 20 mL min ^−1^ and the temperatures of the injector and oven were 110 and 90 °C. Each container was sampled with two technical replicates.

The degree of fruit chilling injury was evaluated with the internal browning (IB) index according to our previous report [46]. Fruits were classified as four scales by the area of browning flesh surface as follows, scale 0 = 0% flesh surface area browned, 1 = 1–25% area affected, 2 = 26–50% area browned, 3 = 51–75% area affected and 4 = 76–100% area browned. The calculation was taken by the following formula: IB index = 100% × Σ [(internal browning scale) × (fruit numbers at that internal browning scale)]/[4 × total number of fruits evaluated].

### 4.3. Determination of Aroma Volatile by HS-GC-MS

The aroma volatiles of peach fruit flesh were collected and determined according to the previous study with some modifications [56]. Peach fruit flesh tissues were grounded into powder in liquid nitrogen and 5 g powder were immediately transferred into a 20 mL vials containing 3 mL 200 mM ethylene diamine tetraacetic acid (EDTA) and 3 mL 20% CaCI_2_, and 30 µL 2-Octanol (0.8 mg × mL^−1^) was added as internal standard. After sealing, the vials were transferred to a solid-phase micro-extraction (SPME) autosampler (Combi PAL, CTC Analytics, Agilent Technologies, Palo Alto, USA) for volatile compounds collection. The concentration of the aroma volatiles was concentrated by a SPME fiber covered with 65 μm of polydimethylsiloxane and divinylbenzene (PDMS-DVB) (Supeclo Co., Bellefonte, PA, USA). After sampling, the determination was taken by an Agilent 7890 N gas chromatography coupled with an Agilent 5975 C mass spectrometer. The aroma volatiles were separated by a DB-WAX capillary column (30 m × 0.25 mm i.d. × 0.25 µm film thickness; J & W Scientific, Folsom, CA, USA). Helium was used as a gas with flow rate of 1.0 mL min^−1^. The temperature initially started at 40 °C and was increased to 100 °C by 3 °C min^−1^ and then to 245 °C at 5 °C min^−1^. The column effluent was ionized by electron ionization (EI) at an energy of 70 eV with a transfer temperature at 250 °C and a source temperature of 230 °C. Volatile compounds were identified by comparing the retention time of authentic standards and their electron ionization mass spectra with the NIST Mass Spectral Library (NIST-08). Quantification of volatiles was performed using the peak area of the internal standard as a reference based on total ion chromatogram (TIC).

### 4.4. Sensory Analysis

To test the effects of CA treatment on peach fruit sensory quality of aroma and flavor, a panel test was performed as previously reported [4]. Briefly, uniform slices of fruit flesh were placed in black soufflé cups with lids that were labeled randomly with three-digit coded numbers. Peach fruits of CA treatment and control were presented to 30 untrained panelists alternatively. Firstly, they had to smell the fruits when the lids were opened and gave a score to the preference of the peach odor. After smelling, panelists were asked to taste the fruit and indicate which one had more flavor and gave scores for the intensity. The time interval between sensory evaluation and cutting fruit were kept within one hour.

### 4.5. RNA Extraction and Real-Time Quantitative PCR Analysis

The total RNA was extracted from approximately 3.0 g frozen peach fruit flesh with CTAB method as described in our previous report [46]. Firstly, the genomic DNA contaminating was removed by RNase-free Dnase I (Thermo Scientific, MBI Fermentas, Burlington, ON, Canada). Then, approximately 2.0 µg RNA was used to synthesized the first-strand cDNA with RevertAidTM Premium Reverase Transcriptase (Thermo Scientific, MBI Fermentas, Burlington, ON, Canada) as manufacturer’s protocol. Quantitative reverse-transcription PCR (RT-qPCR) were analyzed with the following temperature program: initiated at 95 °C for 3 min, followed by 42 cycles at 95 °C for 10 s and 60 °C for 30 s. To normalize small differences of the template amounts, *PpTEF2* was used as the internal control [57]. Results produced by RT-qPCR were expressed as a ratio compared to the initial harvest fruit, which was set as 1. At least three biological replicates for each sample were prepared. All the oligonucleotide primers were listed in Table S2.

### 4.6. Fatty acid Extraction and Determination

The analysis of peach fruit fatty acids was performed as previously reported [57]. Total fatty acids were extracted from 1 g of frozen peach flesh. Fatty acids were converted to volatile methylated fatty acids using 3 mL of methanol: toluene:H_2_SO_4_ (88:10:2, *v/v/v*). The upper phase after derivatization was transferred into vials with heptane and heptadecanoic acid (C17:0) added as internal standards. The content of fatty acids was analyzed by gas chromatograph Agilent 6890N (Agilent, Santa Clara, CA, USA) combined with a DB-WAX column (0.25 mm, 30 m, 0.25 µm, J&W Scientific, Folsom, CA, USA). The conditioned temperature programs were as follows: initial oven temperature at 50 °C, rapidly elevated to 200 °C at 25 °C min^−1^, increased to 230 °C with 3 °C min^−1^, and held for 3 min. Nitrogen was used as carrier gas with constant-flow rate of 1 mL × min^−1^. Determination of fatty acids were detected with FID and identified by comparison with standards of palmitic acid (C16:0), stearic acid (C18:0), oleic acid (C18:1), linoleic acid (C18:2) and linolenic acid (C18:3). The degree of fatty acids unsaturation was indicated by the DBI calculated by the following formula: DBI = [(3 mol% C18:3) + (2 mol% C18:2)]/[(mol% C16:0) + (mol% C18:0) + (mol% C18:1)].

### 4.7. Soluble Sugar and Organic Acid Analysis

The content of soluble sugars and organic acids were determined as noted in our previous report [10]. Briefly, frozen flesh tissues were grounded into powder in liquid nitrogen, and 100 mg of powder was transferred into plastic centrifuge tubes. Firstly, sugars and acids were extracted by 1.4 mL methanol at 70 °C for 15 min, followed by centrifugation at 11,000× *g* for 10 min. The upper phase was carefully transferred to a new tube and ribitol (2 mg × mL^−1^) was added as an internal standard with a ratio of 1:10 (*v/v*). Secondly, 60 µL fresh methoxylamine hydrochloride (20 mg mL^−1^, dissolved in pyridine) was added to mixture, and shook at 200 rpm at 70 °C for 1.5 h. Then, 40 µL Bis (trimethylsilyl) tri-fluoroacetamide + 1% Trimethylchlorosilane was added and incubated for 30 min. Thirdly, one microliter liquid sample was injected and determined by Agilent 6890 N gas chromatograph (Agilent, Palo Alto, CA, USA) equipped with FID detector. The injection was carried out in split mode with 10:1 split ratio. The separation of the mixtures was performed by a HP-5 column (30 m × 0.25 mm i.d. × 0.25 µm film thickness; J&W Scientific, Folsom, CA, USA). The oven temperature programs were as follows: 100 °C for 1 min, increased to 185 °C with a rate of 2.4 °C min^−1^, increased to 190 °C with a rate of 0.3 °C min^−1^, increased to 250 °C with a rate of 8 °C min^−1^, and then increased to 280 °C with a rate of 4.98 °C min^−1^, and held for 3 min. The temperature of oven, inlet and detector was set as 100, 250 and 280 °C, respectively. Components were identified and quantified by comparison with retention time and standard curves of standards.

### 4.8. Statistical Analysis

The figures were generated by ORIGIN 8.0 (Microcal Software, Inc., Northampton, MA, USA). The significant test of the two-sample was calculated by unpaired Student’s *t*-test, and the significant level of multiple groups of the concentrate of the aroma volatiles in Supplemental Table S1 was tested by Duncan’s test in a one-way analysis of variance (ANOVA) (* *p* < 0.05; ** *p* < 0.01; and *** *p* < 0.001) (SPSS 21.0; SPSS Inc., Chicago, IL, USA). The heatmap, PCA, and PLS-DA analysis of the volatile organic compounds were produced by the online website of MetaboAnalyst 5.0 (https://www.metaboanalyst.ca, accessed on 10 May 2022) according to the protocols in our previous report [58].

## 5. Conclusions

In conclusion, CA treatment could effectively improve the flavor quality by alleviating CI in peach fruit during shelf-life after postharvest cold storage. Peach fruit storage was extended from 14 d to 28 d with little CI symptom under CA treatment. Thus, few physiological disorders such as the inhibition of ethylene emission, the defect of softening capacity, and the internal browning of the flesh were observed in peach fruit under CA treatment during shelf-life after cold storage. CA treatment fruit obtained high consumer acceptability, which was mainly due to the higher accumulation of the aroma volatiles derived from fatty acids, including C6 compounds, three esters and three lactones. The higher expression of the related key genes such as *PpAAT1* under CA treatment responded to the high accumulation of fruity note esters and lactones. In addition, the higher content of sucrose and the elevated fatty acids DBI gave us clues to clarify the metabolic mechanism further.

## Figures and Tables

**Table 1:** The content of organic acids and soluble sugars in peach during cold storage plus three shelf-days at 20 °C.

Compound (mg × g^−1^ FW)	Treatment	0 days	14 days	14 S3	28 days	28 S3
Fructose	CK	14.88	13.43 a	13.94 a	21.18 a	41.80 a
	CA		12.53 a	16.39 a	12.20 b	13.78 b
glucose	CK	19.98	19.45 a	14.63 a	27.88 a	46.29 a
	CA		19.07 a	17.57 a	18.27 b	14.05 b
Sucrose	CK	151.1	155.91 a	151.37 b	127.45 b	104.80 b
	CA		145.40 b	165.61 a	153.35 a	138.20 a
Total sugar	CK	185.95	188.79 a	179.93 b	176.51 a	192.90 a
	CA		177.00 b	199.57 a	183.82 a	166.02 b
Malic acid	CK	6.96	6.5	6.67	5.42	6.38
	CA		5.92	7	5.33	6.92
quinic acid	CK	1.5	1.21	0.88	0.58	0.46
	CA		1.08	0.96	1.04	0.71
Citric acid	CK	2.13	2.54	2	2.21	2.13
	CA		2.5	2.08	2.38	1.67
Total acid	CK	10.58	10.25	9.54	8.21	8.96
	CA		9.5	10.04	8.75	9.29

Notes: All data are taken from three biological replicates, and different letters of two lines represent significant difference at *p* < 0.05.

## Data Availability

Not applicable.

## Supplementary Materials

The following are available online at https://www.mdpi.com/article/10.3390/ijms23137141/s1.
