# Supplementary material for: Changes of Sensory Quality, Flavor-Related Metabolites and Gene Expression in Peach Fruit Treated by Controlled Atmosphere (CA) under Cold Storage
Source: Int J Mol Sci. 2022 Jun 27;23(13):7141. doi: 10.3390/ijms23137141 (PMC9266655; doi:10.3390/ijms23137141)
Supplement: Supplementary file 1 [file ijms-23-07141-s001.zip › ijms-1742821-supplementary/Supplementary Figures.pdf]

Supplementary Figures

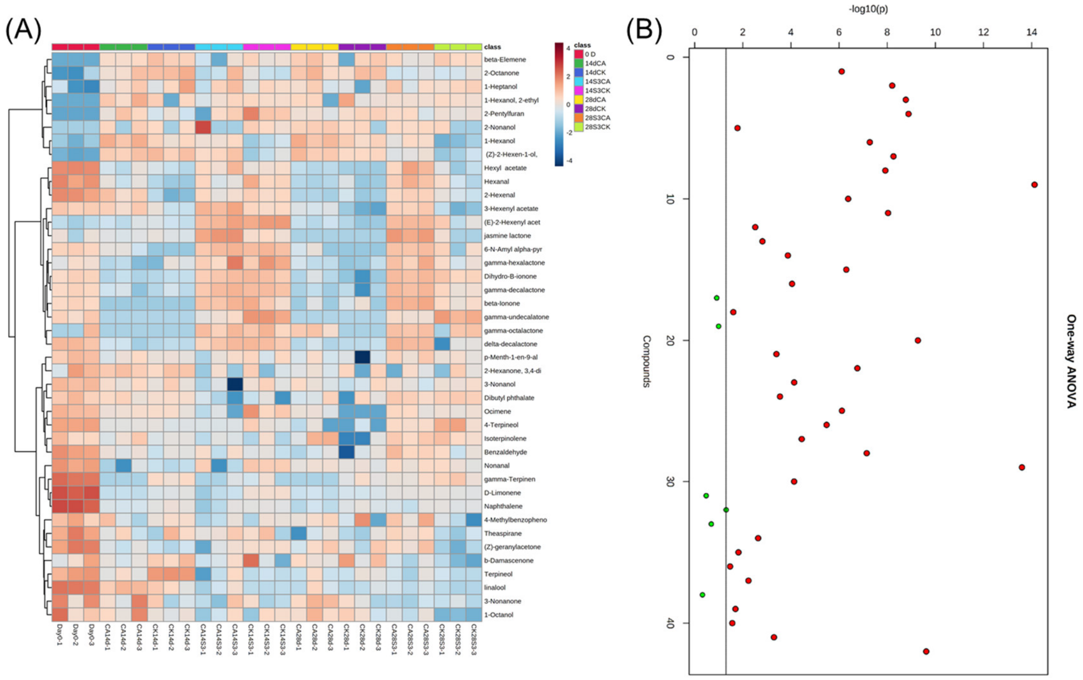

Figure S1. Volatiles of peach fruit under cold storage and shelf-life （**A**), Heatmap of peach fruit volatiles during cold storage and plus shelf-life; (**B**) Statistic analysis of the volatile organic compounds with One-way ANOVA.

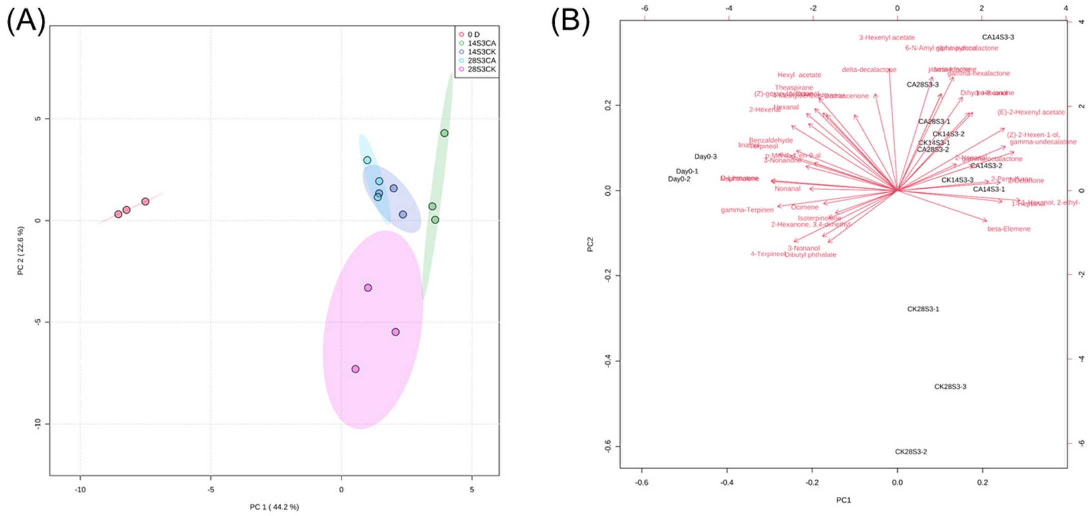

Figure S2. PCA analysis of peach fruit during shelf-life after cold storage. (**A**) PCA score plot of peach fruit VOC; (**B**) Bio-plot of peach fruit VOC with PCA analysis.

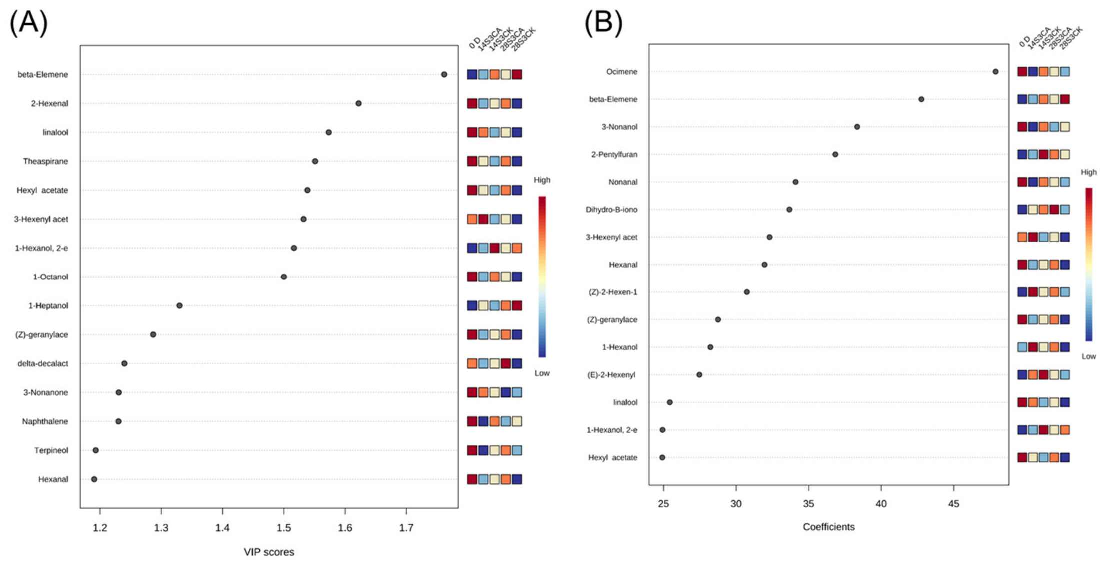

Figure S3. VIP score and coefficients of peach fruit VOC analyzed with PLS-DA during shelf-life after cold storage. (**A**) VIP score of peach fruit VOC; (**B**) Coefficients of peach fruit VOC.

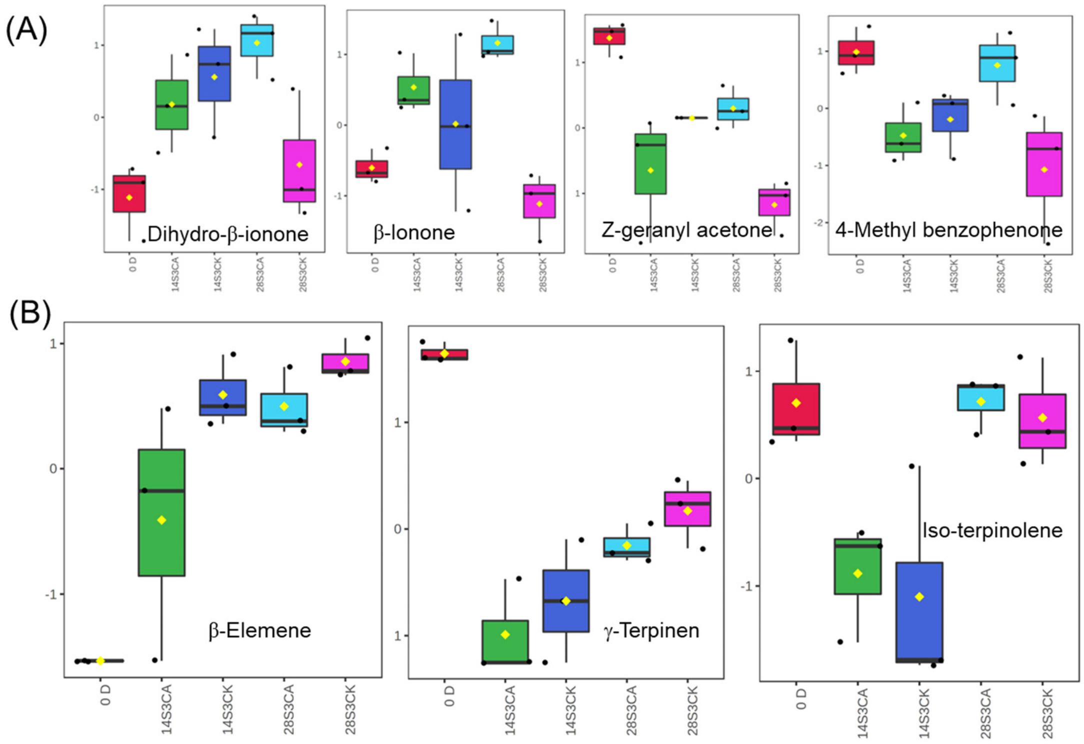

Figure S4. Relative concentration of peach fruit VOC correlated with aroma- and flavor liking. (**A**) Content of volatiles positively correlated with aroma- and flavor liking; (**B**) Content of volatiles negatively correlated with aroma- and flavor liking.

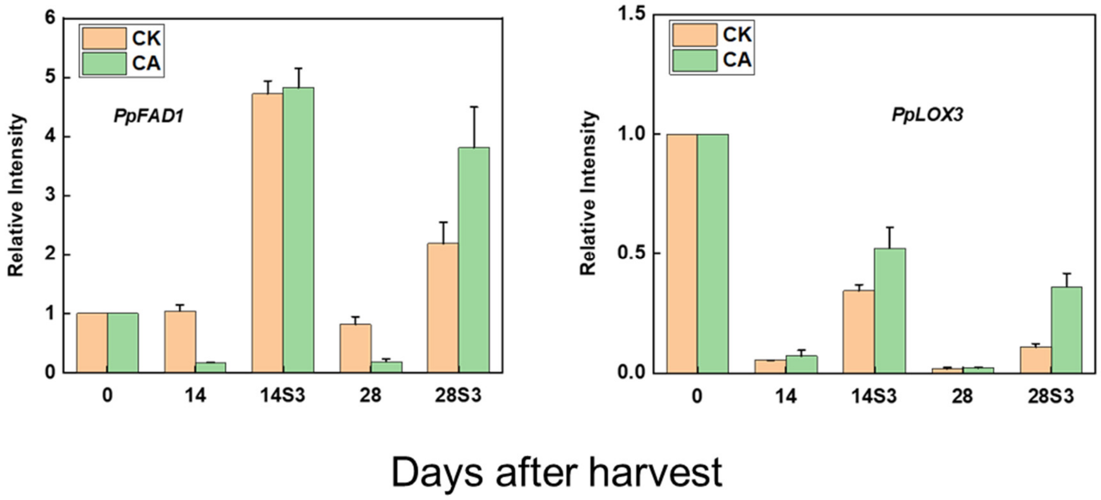

Figure S5. Relative expression of genes from fatty acid oxidation pathway.
